# The Hospital Isolation of Infectious Diseases

**Published:** 1913-06-21

**Authors:** H. Franklin Parsons

**Affiliations:** late of the Medical Department, Local Government Board.


					June 21, 1913. THE HOSPITAL 357
the hospital isolation of infectious diseases.
% H. FRANKLIN PARSONS, M.D., D.P.H., late of the Medical Department, Local Government
Board. /
I.?The History of Isolation.
a a f
The modern system of isolating cases of infec-
tious disease in hospitals provided by the public
authority may be said to date from 1866. The
-London Smallpox Hospital and the London Fever
.^ospital had indeed been established long before
^nafc date, and in several other large towns there
"Were hospitals, often called Houses of Recovery,
'Established and maintained by private charity (with,
it might be, a charge for admission), for cases of
infectious fevers; but, where such special hos-
pitals did not exist, the only accommodation for
?Uch cases prior to 1866 was in the general
.^spitals or in the workhouse infirmaries. In
^ese, fever cases were usually treated, in the same
5ards with those of non-infectious diseases; and,
indeed, many experienced physicians deemed it
, etter to distribute the cases of fever, then chiefly
Jphus, among other patients in general wards,
?father than to treat them in special wards or
gildings; holding that the occasional contracting
of fever by a patient with another disease was, on
tie whole, a less evil than the dangers arising,
oth to the fever patients themselves and to the
? a5, from the concentration of fever cases in
special fever wards. This view was, however, dis-
proved by Dr. Murchison. It had already been
l<:Cogmsed that smallpox cases could not be treated
?ng with others without great danger of the
l^ease spreading.
p |he Sanitary Act of 1866, and subsequently the
ublic Health Act of 1875, now in force, em-
powered local authorities to provide " hospitals or
^rnporary places for the reception of the sick." ?
^ e sick for whom this provision may be made
,.e Hot limited to those suffering from infectious
hav^68' ^ *s chiefly latter that hospitals
.e.been established by public authorities; public
the 1011 *n count*7 favouring the view that
as the non-infectious sick, in so far
t does not fall within the province of the Poor
^a^v, is best left to private charity.
lE Formation of the Meteopolitan Asylums
. Board.
p year or two later, under the Metropolitan
Bo r i"kaw Act, 1867, the Metropolitan Asylums
vis' WaS ^orme(^ ^or the purpose of making pro-
cha?n ^?r reception of certain classes of persons
p^ble to the Poor-Law Unions in the Metro-
' among whom were those suffering from
^0snV?|US ^evers an<^ smallpox. The use of the
first]' ? Prov^e(i by the Asylums Board was at
to **** to paupers, but it was found impossible
restri^^^H ^is limitation, and one by one the
Health 1?-?s Were removed, until by the Public
^Vas m I ^on) Act, 1891, the use of the hospitals
ably Kr6 ^ree inhabitants of London, reason-
?r diphth^V^0 su^er*n? ^rom fever, smallpox,
?th in London and elsewhere the earliest hos-
pitals provided by the local authorities were emer-
gency structures intended to combat some formid-
able epidemic disease, as cholera, smallpox, typ~hus,
or relapsing fever, and were either temporary
buildings hastily erected of wood or other similar
material, or existing buildings converted for use
as hospitals. When the emergency had passed
these temporary hospitals were in some instances
disused; but in other instances they were retained
for the reception of such cases of dangerous in-
fectious disease as might from time to time crop up>
in the district.
In 1882 the Local Government Board issued-
a very valuable report by the late Sir Richard
Thorne Thome " On the Use and Influence of
Hospitals for Infectious Diseases." In this report
it was pointed out that while hospitals for infectious
diseases hurriedly erected under the influence of
panic had often not been ready until the immediate
need for them was past, and had been found ill-
adapted for subsequent use, those, on the other
hand, which had been carefully planned and built
before the time of pressure arose, had proved valu-
able means of defence against the spread of infec-
tious diseases. This report encouraged the erection
of permanent hospitals, and during the next two
decades a large number of districts provided them-
selves with such accommodation, either alone or in
combination with other districts?several County
Councils having promoted comprehensive hospital
schemes for their areas under the Isolation Hos-
pitals Act, 1893.
Popularity of the Isolation Hospital.
Together with the increase in the amount of
hospital accommodation provided by the authorities
there has been an increased disposition on the
part of the public to make use of it. In former
times there was often great reluctance on the
part of parents to allow a child suffering from
infectious illness to be removed to hospital, and
older persons were unwilling to go, so that a,
magistrate's order for removal had sometimes to
be obtained. But with increasing experience on
the part of the public of the advantages of hospital
treatment and of the convenience of the removal of
infectious cases from the household, together with,
the usual abandonment of the practice of making-
a charge for the maintenance of the patient in
hospital, this objection has largely ceased, and
indeed the pendulum has swung so far in the
opposite direction that it is now sometimes com-
plained of by parents as a grievance if their child
when suffering from an infectious disease
cannot be taken into hospital.
There has also, since isolation hospitals were
first instituted, been a great change in the nature of
the cases admitted. As already mentioned, the
earliest hospitals were used chiefly for the more
358 THE HOSPITAL June 21, 1913.
formidable epidemic diseases; but of these, cholera,
typhus and relapsing fever are now extinct, or
almost so, in this country. Smallpox has practi-
cally ceased to be an endemic disease; and when
outbreaks occur it is treated in separate hospitals.
On the other hand, scarlet fever, which at the time
of Sir R. Thorne Thome's report had only rarely
been the immediate cause of hospital provision, not-
withstanding the large mortality which it then
occasioned, has now?although at the present time
a comparatively mild disease?come to be the
principal disease treated in isolation hospitals; the
number of admissions for scarlet 'fever is com-
monly much larger than that for any other disease,
or even for all other diseases; and indeed it has
sometimes happened that a local authority has
reserved its hospital for scarlet-fever cases only,
to the exclusion of other and more formidable
diseases. The other diseases commonly treated
in isolation hospitals are enteric fever and diph-
theria. These were formerly often treated in
general hospitals, but their infectious nature is
now more generally recognised than it was thirty
years ago?it was only in 1889 that diphtheria
became legally admissible into the hospitals of the
Metropolitan Asylums Board?and they are now
relegated to the isolation hospital.
Pbogress Dueing the Past Thirty Years.
Quite recently the hospital treatment of other
infectious diseases, as measles, puerperal fever, and
erysipelas, and also of pulmonary tuberculosis?
diseases formerly thought unsuitable as a rule for
admission into isolation hospitals?has been advo-
cated on the ground of the benefit accruing to the
patient from treatment rather than from that of the
protection afforded to the public by the patient's
isolation. Thus during the past thirty years there
has been a large and progressive increase both in
the total number of cases of infectious disease
admitted into isolation hospitals and in the propor-
tion of admissions to cases occurring. This
increase has been due to several causes; among
which the following are the principal:
1. Through the notification of infectious diseases
and improved sanitary supervision a larger number
of the infectious cases occurring are brought to
knowledge.
2. More ample hospital accommodation has been
provided.
3. A greater range of diseases is admitted.
4. Removal of restriction to paupers, and of dis-
qualifications entailed by use of hospitals.
5. Charges for admission to hospital have been
abolished in London and many other districts, and
are often remitted in others.
6. There is a greater appreciation by the public
of the benefit of hospital treatment and of the con-
venience of getting rid of infectious cases out of the
household.
The following table shows the course of scarlet
fever in London since the coming into force of com-
pulsory notification in 1890, and the opening of the
Asylums Board's hospitals free of cost to all in-
habitants of London suffering from the disease, to
1911. It will be seen that during this period the-
percentage of cases removed to hospital has doubled,,
nine out of every ten notified being now removed,
and that concurrently with this increase there has-
been a great decrease in the mortality from scai'let
fever, which is now only one-fifth of what it was
at the beginning of the period. There has also been
a decrease, but to a less extent and with fluctua-
tions, in the proportion of notified cases to popula-
tion, and a very great decrease in the proportion of
deaths to cases both among the total cases notified
and among the cases admitted to hospital; the pro-
portion is, however, uniformly slightly higher
among the latter than among the former, though the
difference diminishes as the proportion of removals
to notified cases increases.
Table I.?Scarlet Fever in London.
Year
1890
1891
1892
1893
1894
1895
1896
1897
1898
1899
1900
1901
1902
1903
1904
1905
1906
1907
1908
1909
1910
1911
Notifications!
per 1,000
Population
Deaths
per 1,000
Population I
Percentage
of Deaths
to Notifica-
tions
to
Percentage
of Cases
removed to
Hospital to
Notifications!
42.8
83.8 ?
84.5 ??
88.6 IS
88.5 [o
89.4 g>
90.9 g
90 2 g
88.9/ %
89.2
of Deat^is.
in Hospitf1?
to Cases
Admitted
7.9
6.7\
7.3
6.1
5.9
IW
4.1
4.1
2.61 _
3.0' <
3.1
3.4
3.3
2.9
28
2.6
23, 5
2.4' <
1.9

				

